# The complete chloroplast genome sequence of *Sinojackia huangmeiensis* (Styracaceae)

**DOI:** 10.1080/23802359.2020.1714508

**Published:** 2020-01-20

**Authors:** Hongjin Dong, Hongyu Wang, Yanling Li, Jiaojun Yu

**Affiliations:** a Hubei Key Laboratory of Economic Forest Germplasm Improvement and Resources Comprehensive Utilization, Huanggang Normal University, Huanggang, China;; b Hubei Collaborative Innovation Center for the Characteristic Resources Exploitation of Dabie Mountains, Huanggang Normal University, Huanggang, China

**Keywords:** *Sinojackia huangmeiensis*, complete chloroplast genome, Styracaceae, phylogeny

## Abstract

*Sinojackia huangmeiensis* J. W. Ge & X. H. Yao is a member of the genus *Sinojackia* endemic to central and east China. Here we assembled and annotated the complete chloroplast (cp) genome. It is 158,758 bp in length and encodes 84 protein-coding genes, 37 transfer RNA (tRNA) genes and eight ribosomal RNA (rRNA) genes. The Maximum likelihood phylogenetic analysis confirm that *S. huangmeiensis* is a closely related but another different species to *S. sarcocarpa*.

*Sinojackia* is a small genus with ca. eight species endemic to central and east Asia (Hu 1928; Zhang et al. 2012)*. S. huangmeiensis* is one member of this genus, which is only found in the type locality till now (Yao et al. 2007). Only about 400 individuals are reported in the small area (Luo et al. 2016), but it’s hard to propagate itself. Own white flower and scopperil-shaped fruit, and special systematic position, this species is of important value in horticulture and phylogeny (Luo et al. 2016; Zhao et al. 2016). In present study, the completed chloroplast genome sequence of *S. huangmeiensis* is reported contributing to conservation of this species and providing significant information for the phylogeny of Styracaceae.

Genomic DNA was extracted from leaves of a seedling of *S. huangmeiensis* from Qianlin Villadge, Xiaxin Town, Huangmei County in Hubei Province, China (116°00′47.36″E, 29°59′31.91″N; 20 m in altitude, near the lake, *Dong* et al.*-HGNU-0289*, 2019. 4. 18; HGTC). Those leaves were stored in the refrigerator at −80 °C. The total genomic DNA was isolated according to a modified CTAB method (Doyle 1987). Total genome DNA of *S. huangmeiensis* was sequenced by Illumina Hiseq 2500 Sequencing System (Illumina, Hayward, CA) to construct the shotgun library. About 10 Gb pair-end (150 bp) raw short sequence data were obtained. The low quality sequences were filtered out Using CLC Genomics Workbench v8.0 (CLC Bio, Aarhus, Denmark) and then reconstructed the chloroplast genome by using MITObim v1.8 (University of Oslo, Oslo, Norway; Kaiseraugst, Switzerland) (Hahn et al. 2013). The complete chloroplast genome of *S. huangmeiensis* was annotated in Geneious R9 (v9.0.2) (Matthew et al. 2012) and online program Chloroplast Genome Annotation, Visualization, Analysis, and GenBank Submission (CPGAVAS) (Institute of Medicinal Plant Development, Chinese Academy of Medical Sciences and Peking Union Medical College, Beijing, China) (Liu et al. 2012) and then submitted to GenBank (accession no. MN694844).

The size of chloroplast genome of *S. huangmeiensis* is 158,758 bp, including a large single-copy (LSC) region of 88,023 bp and a small single-copy (SSC) region of 18,555 bp separated by a pair identical inverted repeat regions (IRs) of 26,090 bp each. A total of 139 genes were successfully annotated containing 84 protein-coding genes, 37 tRNA genes and 8 rRNA genes. GC content of the whole genome, IRs, LSC and SSC regions are 37.2%, 43.0%, 35.2% and 30.5%, respectively. GC content of IRs region is the highest. 17 genes contain one intron, while 2 genes have two introns. The complete chloroplast genome sequence of *S. huangmeiensis* and other species from Styracaceae were used to construct phylogenetic tree (Figure 1). Take the plastome of Euryodendron excelsum (NC_039178) as an out-group, a maximum likelihood analysis was performed with RAxML version 8 program (Alexandros 2014) using 1000 bootstrap. The ML tree shows all the published plastome of *Sinojackia* were grouped into a clade and the phylogenetic relationship was consistent with the previously-published phylogeny (Yao et al. 2008; Cai et al. 2018; Fan et al. 2019). The results show that *S. huangmeiensis* was most close to *S. carcocarpa*.

**Figure 1.**
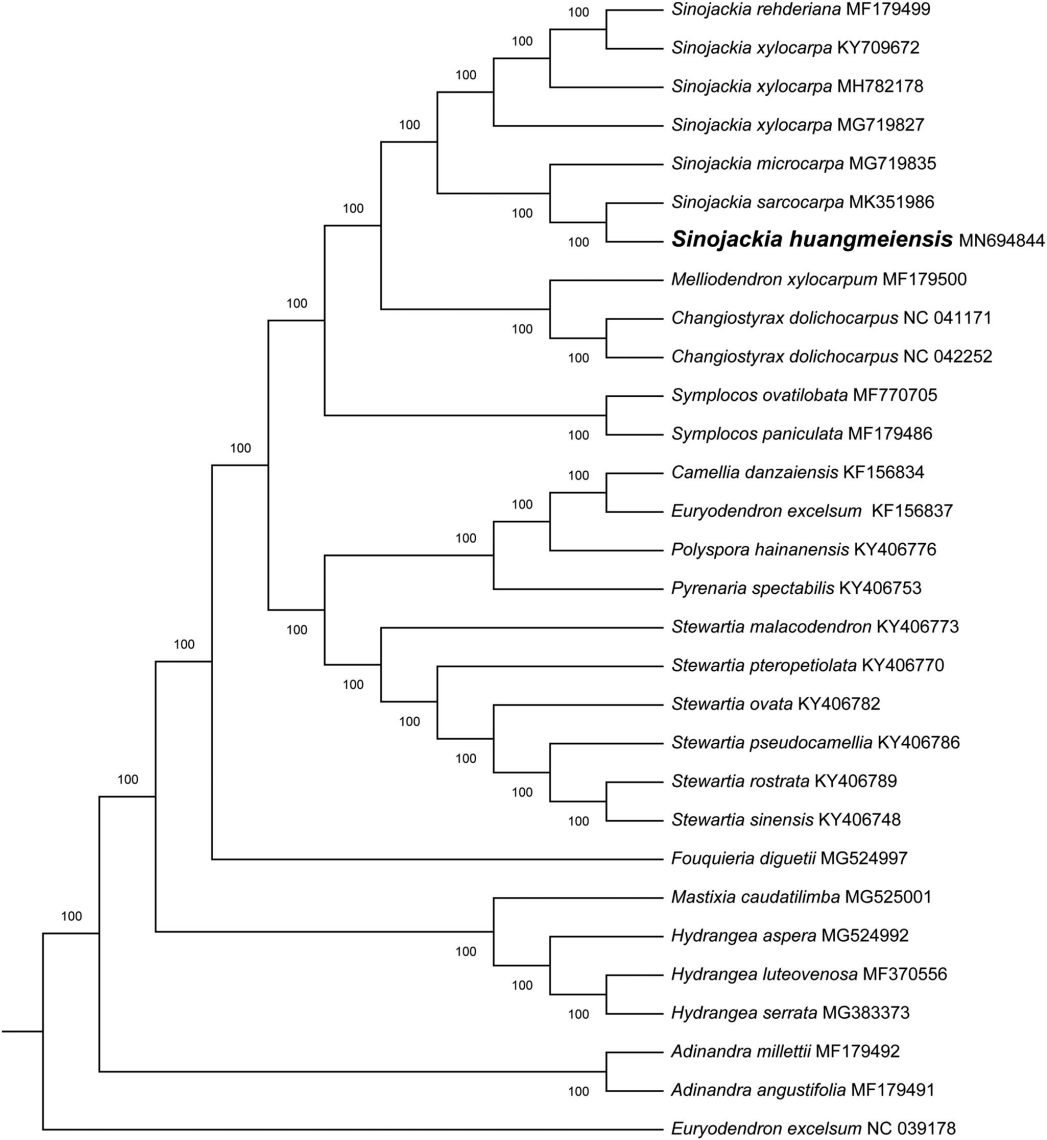
Maximum likelihood phylogenetic tree for *Sinojackia huangmeiensis* based on 30 complete chloroplast genomes. The number on each node indicates bootstrap support value.
